# Exploring the relationship between psychological capital, emotional intelligence, psychological safety, and knowledge sharing among E-commerce practitioners

**DOI:** 10.3389/fpsyg.2024.1472527

**Published:** 2024-10-28

**Authors:** Xuan Zhang, Ziqing Xu

**Affiliations:** ^1^Business School, Huaiyin Normal University, Huaian, China; ^2^Business School, Guangdong Ocean University, Yangjiang, China

**Keywords:** psychological capital, emotional intelligence, psychological safety, knowledge sharing, E-commerce practitioners

## Abstract

**Introduction:**

This study explores the intricate relationships among psychological capital, emotional intelligence, psychological safety, and knowledge sharing among e-commerce practitioners. Grounded in social exchange theory, the research aims to fill a gap in the literature by focusing on the psychological and emotional factors influencing knowledge sharing in a fast-paced and highly competitive industry.

**Methods:**

This study used snowball and purposive sampling to collect 439 valid online questionnaires from e-commerce professionals in Guangdong, Zhejiang, and Jiangsu, China. The survey, conducted from October to November 2023, explored the relationships between psychological capital, emotional intelligence, psychological safety, and knowledge sharing. Data were analyzed using structural equation modeling (SEM) with AMOS 26.0.

**Results:**

The structural path model revealed significant positive correlations between psychological capital and both emotional intelligence (*β* = 0.494, *p* < 0.001) and psychological safety (*β* = 0.297, *p* < 0.001). Emotional intelligence was significantly positively related to psychological safety (*β* = 0.513, *p* < 0.001) and knowledge sharing (*β* = 0.452, *p* < 0.001). Psychological safety was also positively correlated with knowledge sharing (*β* = 0.311, *p* < 0.001). Bootstrap analysis indicated that emotional intelligence and psychological safety significantly mediate the relationship between psychological capital and knowledge sharing (standardized indirect effect = 0.394, *p* < 0.01).

**Discussion:**

Based on the significant findings of this study, a key recommendation is to implement targeted interventions aimed at enhancing psychological capital, emotional intelligence, and psychological safety among e-commerce practitioners. Specifically, developing training programs focused on building resilience, self-efficacy, hope, and optimism can improve employees’ psychological capital. Additionally, workshops designed to enhance emotional intelligence and create a culture of psychological safety can encourage open communication and trust, thereby promoting knowledge sharing.

## 1 Introduction

Over the past decade, the e-commerce industry has experienced unprecedented growth, transforming the way businesses operate and consumers shop (Zenkina, 2022). This rapid expansion is driven by technological advancements, increased internet penetration, and consumers’ preferences for convenience and a wider variety of products (Thakur, 2021; Costa and Castro, 2021). In 2023, global retail e-commerce sales reached an estimated $5.8 trillion. Projections indicate a 39% growth in this figure over the coming years, with expectations to surpass $8 trillion by 2027 (Statista, 2023). China, in particular, has seen exponential growth in online shopping activities due to its large population and rapid technological advancements (Jiang et al., 2023). With giants such as Alibaba, Tictok, and Temu leading the way, China boasts the world’s largest e-commerce market (Qin et al., 2023). According to Statista’s Digital Market Outlook, the live streaming e-commerce market in China reached nearly 5 trillion yuan in 2023, up from 420 billion yuan in 2019, and is forecast to surge to 8.16 trillion yuan by 2026 (Statista, 2023). Additionally, data from the National Bureau of Statistics of China revealed that the number of e-commerce industry employees doubled from 33 million in 2018 to 73.05 million in 2023 (China NBoSo, 2023). Both in terms of industry development and demand, the e-commerce sector requires a vast pool of talent to sustain its growth (Ning, 2017). Enhancing the skills and capabilities of e-commerce industry professionals is a significant challenge that e-commerce companies currently face (Al-Tayyar et al., 2021; Chin et al., 2023).

Much of the existing research on e-commerce can be categorized into two primary perspectives: the consumer perspective and the enterprise perspective. From the consumer perspective, studies have focused on increasing purchase intentions and enhancing user experiences (Bylok, 2022; Chen and Yang, 2021; Wang et al., 2023). Researchers have explored various strategies to boost consumer engagement, satisfaction, and loyalty. For instance, personalized marketing, which tailors product recommendations based on consumer behavior, has been shown to significantly increase purchase intentions (Pappas et al., 2017). Additionally, user-friendly website design and efficient customer service have been highlighted as critical factors in enhancing the overall shopping experience and retaining customers (Sangwan et al., 2022; Tarkang et al., 2022). On the other hand, the enterprise perspective emphasizes building and optimizing e-commerce business models, improving supply chain efficiency, and integrating online and offline channels to create a seamless omni-channel experience (He, 2022; He et al., 2022). Studies in this domain aim to enhance company performance and competitiveness by addressing key operational challenges and leveraging technological innovations (Suali et al., 2024). For example, research has shown that optimizing supply chain management through advanced analytics can significantly reduce costs and improve delivery times, thereby enhancing customer satisfaction (Qin and Liu, 2022). Furthermore, the integration of online and offline channels, known as omni-channel retailing, has been identified as a crucial strategy for providing a consistent and comprehensive shopping experience, which is vital for maintaining a competitive edge in the market (Hazen and Byrd, 2012).

However, despite the extensive research on consumer behavior and enterprise strategies, there is relatively little research focused on the perspective of e-commerce practitioners. In the e-commerce sector, practitioners face significant pressure and challenges due to the rapid development and highly competitive nature of the industry (Chen et al., 2020; Wang et al., 2018). They need to master complex technical and market knowledge while also dealing with frequent changes and high-intensity work environments (Kovács and Keresztes, 2022; Rahayu and Day, 2017).

For e-commerce enterprises, which typically involve multiple departments such as marketing, technology, and customer service, effective communication and interaction among employees are crucial (Wang et al., 2022). Knowledge sharing, as a key organizational capability, can break down departmental barriers, promote cross-departmental collaboration, and ensure coordinated efforts across various functions, thereby enhancing overall business performance (Xu and Suntrayuth, 2022). Existing research has also confirmed that knowledge sharing among employees is essential for fostering innovation and maintaining competitive advantage. Effective knowledge sharing can improve organizational performance and establish a culture of sustained competitive advantage (Arsawan et al., 2022). However, current research on how to promote knowledge sharing among e-commerce practitioners remains neglected.

To address this research gap, this study is based on social exchange theory (SET), which provides a valuable framework for understanding how to enhance knowledge sharing among e-commerce practitioners. SET posits that social behavior is the result of an exchange process aimed at maximizing benefits and minimizing costs (Park et al., 2015). In an organizational context, SET highlights the importance of reciprocity, trust, and mutual benefit in promoting cooperative behaviors such as knowledge sharing (Yan et al., 2016). Previous research has shown that emotional intelligence and psychological safety have significant positive effects on knowledge sharing (Malik, 2021a). Psychological capital, which includes self-efficacy, hope, resilience, and optimism, helps practitioners better cope with work-related stress, enhancing their job performance and satisfaction (Qian et al., 2020). These psychological and emotional factors not only impact their work performance and satisfaction but also have a profound influence on their knowledge sharing behavior (Xu et al., 2023). By applying SET, this study aims to explore the relationships between psychological capital, emotional intelligence, psychological safety, and knowledge sharing among e-commerce practitioners. This approach seeks to fill the current empirical research gap and provide insights that can help e-commerce companies foster a more collaborative and innovative work environment.

Therefore, the primary objectives of this study are threefold. First, it aims to investigate the impact of psychological capital, emotional intelligence, and psychological safety on knowledge-sharing behaviors among e-commerce practitioners—an area that has received limited attention in current literature despite the critical role of knowledge sharing in organizational success. Second, this study seeks to validate the applicability and effectiveness of SET in explaining the relationships between these psychological factors and knowledge sharing within the e-commerce sector. While SET has been widely applied in other organizational contexts, its use in the specific context of e-commerce practitioners is underexplored, representing a key gap in existing research. Third, the study aims to provide actionable recommendations for e-commerce companies to foster a supportive environment that promotes knowledge sharing, thereby addressing the practical challenge of enhancing employee collaboration in a highly competitive industry.

The significance of this research lies in its academic and practical contributions. Academically, it addresses the current gap by offering empirical evidence on how psychological factors influence knowledge-sharing behaviors among e-commerce practitioners, enriching the application of SET in this unique context. This study also fills the gap by focusing on the internal dynamics of e-commerce organizations, which have been largely neglected in favor of research centered on consumer and enterprise strategies. Practically, the findings will provide valuable strategies for e-commerce companies to enhance their organizational performance and innovation capacity by improving psychological capital, emotional intelligence, and psychological safety, ultimately fostering a culture of knowledge sharing that drives overall success and maintains a competitive edge in the market.

The following sections are structured as follows: Section 2 presents the hypotheses and conceptual models. Section 3 introduces data collection and data analysis methods. Section 4 describes the results of the data analysis and tests the hypotheses. Section 5 discusses the theoretical implications, practical implications, and limitations with directions for future studies.

## 2 Literature review

### 2.1 Social exchange theory

SET has emerged as a foundational framework for understanding interpersonal relationships and behaviors within organizational contexts. Developed by George Homans in the 1950s and further expanded by Peter Blau, SET posits that social behavior results from an exchange process where individuals seek to maximize benefits and minimize costs in their interactions (Oparaocha, 2016). This theoretical lens emphasizes that relationships are formed and maintained based on the perceived value of the exchanged resources, which may include tangible assets, emotional support, or knowledge (Yan et al., 2016; Schoenherr et al., 2015). A core concept of SET is reciprocity, which suggests that individuals engage in mutually beneficial exchanges, fostering a sense of obligation and trust (Lai et al., 2014). This is particularly relevant in the context of knowledge sharing, as employees are more likely to share knowledge when they believe their contributions will be reciprocated by colleagues and the organization (Kim et al., 2015).

Research has increasingly applied SET to explore knowledge sharing across various organizational contexts. For instance, employees are more likely to share knowledge when they perceive a strong sense of reciprocity and support from their colleagues and organization (Kim et al., 2017). This highlights that when individuals feel their contributions will be valued and rewarded, their engagement in knowledge sharing increases significantly. Moreover, social exchange relationships often foster an environment of trust and collaboration, which are essential for effective knowledge sharing. Trust can be categorized into two dimensions: cognitive trust, based on the reliability of others, and affective trust, based on emotional bonds (Park et al., 2015; Wu and Lee, 2017). Both dimensions are crucial in promoting a culture of knowledge sharing, as they encourage individuals to openly share their expertise without fear of negative repercussions.

In the context of e-commerce practitioners, applying SET provides valuable insights into enhancing knowledge sharing practices. Understanding that employees are motivated by perceptions of reciprocity and support can guide organizations in fostering an environment that prioritizes psychological safety and emotional intelligence. As e-commerce operates in a fast-paced and competitive environment, promoting a culture of trust and collaboration is vital for driving innovation and performance. This study aims to fill the gap in existing research by exploring the relationships between psychological capital, emotional intelligence, psychological safety, and knowledge sharing among e-commerce practitioners. By leveraging SET, e-commerce companies can better understand and facilitate the conditions under which their employees are most likely to share knowledge, thereby enhancing overall organizational effectiveness.

### 2.2 Effect of psychological capital on emotional intelligence

Psychological capital is a core construct in positive organizational behavior, encompassing self-efficacy, hope, resilience, and optimism (Peng et al., 2022). These components are crucial for individuals’ overall psychological well-being and their ability to thrive in challenging work environments. Psychological capital influences various positive outcomes, such as job satisfaction, performance, and organizational commitment (Luthans et al., 2008). In the context of e-commerce practitioners, who often face high levels of stress and dynamic work conditions, psychological capital can be particularly significant.

The relationship between psychological capital and emotional intelligence shows that individuals with high levels of psychological capital are more likely to exhibit higher because the positive psychological resources inherent in psychological capital enhance emotional regulation and interpersonal skills (Zhou et al., 2022). For example, self-efficacy, a component of psychological capital, is associated with greater confidence in managing emotional challenges, while optimism and hope contribute to a positive outlook that facilitates better emotional interactions (Jing et al., 2022; Narayanasami et al., 2024). A meta-analysis found that psychological capital is positively related to emotional intelligence across various contexts (Sharma and Barmola, 2023). This relationship suggests that enhancing psychological capital can lead to improvements in emotional intelligence, which is crucial for e-commerce practitioners who must navigate complex emotional landscapes in their interactions with customers, colleagues, and stakeholders. Therefore, the following hypothesis is presented:

*Hypothesis 1 (H1)*: Psychological capital is positively correlated with emotional intelligence.

### 2.3 Effect of psychological capital on psychological safety

Psychological safety, defined as the shared belief that it is safe to take interpersonal risks in a work environment, is crucial for fostering an atmosphere where employees feel comfortable expressing their thoughts, questions, and concerns without fear of negative consequences (Bergheim et al., 2015). Psychological safety is associated with enhanced team performance, learning behavior, and innovation (Elliott and Fry, 2021). It enables employees to engage openly and collaborate effectively, which is vital in dynamic and collaborative e-commerce environments.

High Psychological capital individuals are more likely to experience psychological safety because their positive psychological resources empower them to interact confidently and constructively with colleagues (He et al., 2019). For instance, self-efficacy can boost confidence in participating in group discussions, while resilience helps individuals recover from setbacks in team interactions (Saleem et al., 2022). Optimism and hope contribute to a supportive and trusting atmosphere, encouraging open communication and risk-taking (Stratman and Youssef-Morgan, 2019). Therefore, the following hypothesis is presented:

*Hypothesis 2 (H2)*: Psychological capital is positively correlated with psychological safety.

### 2.4 Effect of emotional intelligence on psychological safety

Emotional intelligence refers to the ability to recognize, understand, and manage one’s own emotions as well as the emotions of others (Zhou et al., 2020). It encompasses key components such as self-awareness, self-regulation, motivation, empathy, and social skills. These components enable individuals to navigate social complexities, foster positive relationships, and make informed decisions based on emotional cues. Emotional intelligence is particularly important in work environments that require high levels of interaction and collaboration, such as in e-commerce.

Individuals with high emotional intelligence are more adept at understanding and managing their own emotions, which helps them to remain calm and composed in stressful situations. This emotional regulation fosters a sense of security and trust among team members, as they perceive the emotionally intelligent individual as stable and reliable (Shankar and Tewari, 2023). Moreover, individuals with high emotional intelligence are better at empathizing with others, which enables them to build stronger interpersonal connections and promote a supportive team environment (Munawar et al., 2024). Research suggests that leaders with high emotional intelligence can create a more psychologically safe environment for their teams by demonstrating empathy, effective communication, and emotional support (Gheorghe et al., 2024). These leaders are better equipped to address and mitigate conflicts, ensuring that team members feel heard and valued. Therefore, the following hypothesis is presented:

*Hypothesis 3 (H3)*: Emotional intelligence is positively correlated with psychological safety.

### 2.5 Effect of emotional intelligence and psychological safety on knowledge sharing

Knowledge sharing is a critical process within organizations that fosters innovation, improves performance, and sustains competitive advantage (Malik, 2021a). It involves the exchange of information, skills, and expertise among employees, enabling organizations to leverage collective knowledge for problem-solving and decision-making. In the context of e-commerce, where rapid changes and high competition are prevalent, effective knowledge sharing is essential for organizational success (Malik, 2021b).

Emotional intelligence plays a significant role in promoting knowledge sharing. Individuals with high emotional intelligence are better at managing their own emotions and understanding others’ emotions, which facilitates open communication and trust (Malik, 2022). These emotional competencies help create a collaborative environment where employees feel comfortable sharing their knowledge and expertise. Research indicates that employees with high emotional intelligence are more likely to engage in knowledge sharing because they can effectively navigate social interactions, manage conflicts, and build strong interpersonal relationships (Naz and Li, 2019; Jamshed and Majeed, 2019).

Psychological safety, defined as the belief that one will not be punished or humiliated for speaking up with ideas, questions, concerns, or mistakes, is another crucial factor influencing knowledge sharing (Qian et al., 2020; Siemsen et al., 2009). A psychologically safe environment encourages employees to take interpersonal risks without fear of negative consequences, thereby promoting open dialog and the free exchange of information (Wang et al., 2018). In teams with high psychological safety, members are more likely to share their knowledge because they trust that their contributions will be valued and respected. This positive correlation between psychological safety and knowledge sharing is supported by several studies, which highlight that psychological safety significantly enhances knowledge sharing among team members (Yang et al., 2023). Therefore, the following hypothesis is presented:

*Hypothesis 4 (H4)*: Emotional intelligence is positively correlated with positive knowledge sharing.

*Hypothesis 5 (H5)*: Psychological safety competence is positively correlated with positive knowledge sharing.

### 2.6 Mediating roles of emotional intelligence and psychological safety

Although there is currently no literature directly studying the mediating relationships among psychological capital, emotional intelligence, psychological safety, previous studies have emphasized the importance of psychological safety and emotional intelligence in organizational settings. For instance, research has explored the mediating role of psychological safety in the relationship between leadership styles and employee engagement, confirming that psychological safety significantly contributes to improved employee engagement (Quansah et al., 2023). Similarly, studies have shown that emotional intelligence mediates the relationship between leader support and knowledge sharing, demonstrating that high emotional intelligence enables better handling of workplace challenges and promotes positive interactions (Shariq et al., 2019). These findings provide a solid foundation for investigating the mediating roles of emotional intelligence and psychological safety in the context of e-commerce practitioners.

**Figure 1:**
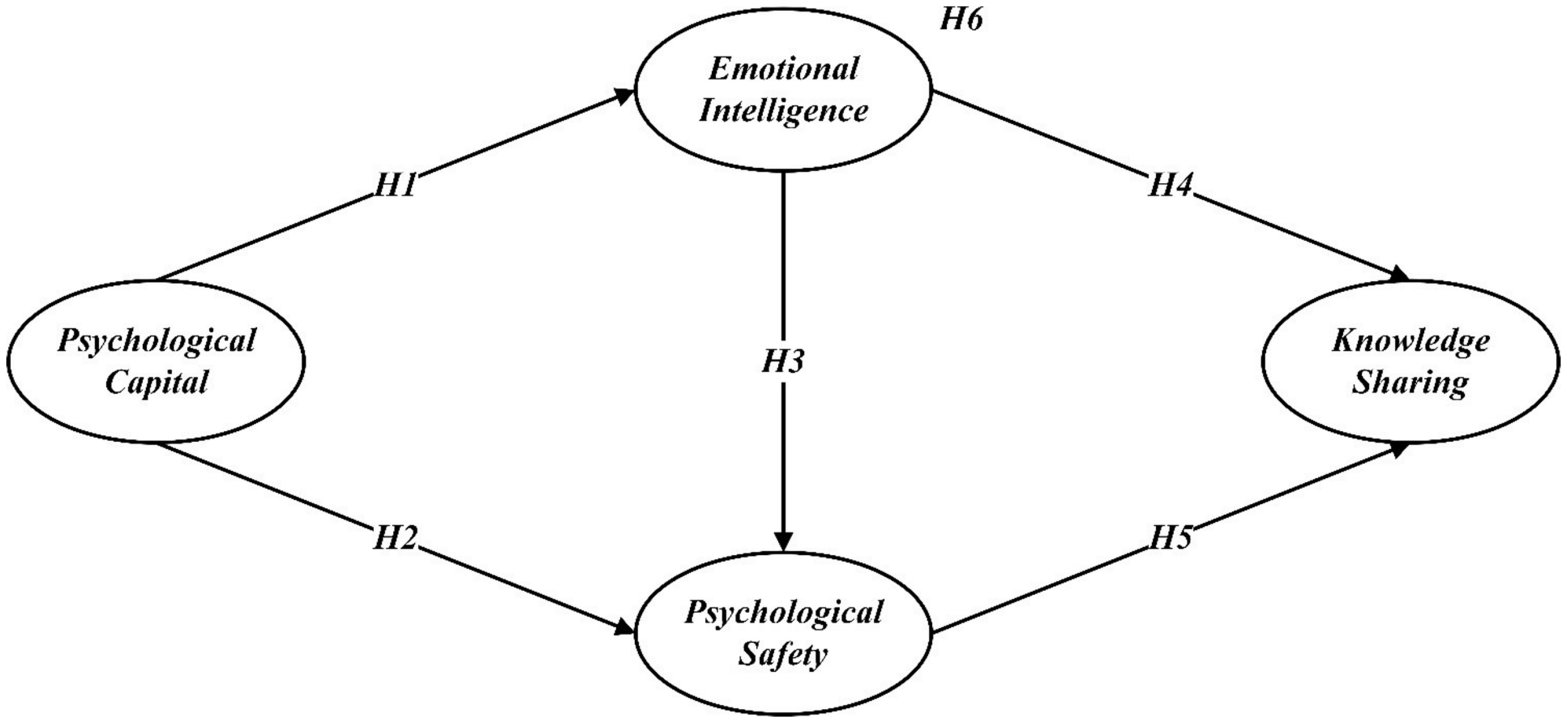
The hypothesized model.

Emotional intelligence is an individual’s ability to recognize, understand, and manage their own emotions as well as the emotions of others (Zhou et al., 2020). Psychological capital, which includes self-efficacy, hope, resilience, and optimism, can enhance an individual’s emotional intelligence by providing the psychological resources necessary for effective emotional regulation and interpersonal interactions (Edmondson, 1999). Individuals with high psychological capital are more likely to exhibit higher emotional intelligence, which in turn facilitates better communication, trust, and collaboration among employees, leading to increased knowledge sharing (Siemsen et al., 2009). Furthermore, individuals with high psychological capital are more resilient, optimistic, and confident, which helps create a psychologically safe environment where employees feel comfortable sharing their ideas and knowledge without fear of negative consequences (He et al., 2019). Psychological safety thus provides a supportive context that encourages open dialog and collaboration, further enhancing knowledge sharing (Wu et al., 2022). Thus, this study aims to explore the mediating roles of emotional intelligence and psychological safety in the relationships among psychological capital and knowledge sharing (Figure 1). Therefore, the following hypothesis is presented:

*Hypothesis 6 (H6)*: Emotional intelligence and psychological safety mediate the relationship between psychological capital and knowledge sharing.

## 3 Method

### 3.1 Participants

Researchers used both snowball and purposive sampling techniques to gather 481 online questionnaires from individuals working in e-commerce. Following the exclusion of 42 questionnaires that were invalidated due to identical responses or overly rapid completion, a total of 439 questionnaires remained valid. This resulted in an effective response rate of 91%. Demographic details of the e-commerce professionals who participated in this study are displayed in Table 1.

**Table 1:** Overview of participants.

Profiles	Frequency	Proportion
Gender		
Male	235	53.5
Female	204	46.5
Age		
≤28	287	65.4
29–35	86	19.6
≥35	66	15.0
Province		
Guangdong	141	32.1
Zhejiang	201	45.8
Jiangsu	97	22.1
Job category		
Customer service representatives	137	31.2
Logistics coordinators	123	28.0
Sales support personnel	179	40.8
Monthly income		
≤10,000 CNY	296	67.4
10,001–20,000 CNY	69	15.7
≥20,001 CNY	74	16.9

### 3.2 Procedure

This study targeted e-commerce practitioners as its research subjects. Researchers contacted grassroots employees on e-commerce platforms in Guangdong, Zhejiang, and Jiangsu, China, including customer service representatives, logistics coordinators, and sales support personnel. The purpose and significance of the study were explained to them, focusing on exploring the relationship between psychological capital, emotional intelligence, psychological safety, and knowledge sharing. Using snowball and purposive sampling methods, these grassroots employees were asked to distribute the questionnaire to their colleagues within the industry. The survey was conducted from October to November 2023, ensuring the anonymity of all respondents.

### 3.3 Measures

The questionnaire was the consideration of SET, which serves as the theoretical foundation for this study. SET emphasizes reciprocal behaviors, trust, and mutual benefits in promoting cooperative actions such as knowledge sharing. The questionnaire consisted of four main variables, each corresponding to one of the core constructs.

Psychological capital reflects how these factors influence respondents’ ability to handle challenges in their work environment. The Psychological Capital Scale developed by Lorenz et al. (2016), featuring four items such as “I can think of many ways to reach my current goals,” was employed to measure psychological capital. The emotional intelligence section was based on widely used measures that capture respondents’ ability to recognize, understand, and manage emotions in themselves and others, which is critical for fostering collaboration. Emotional intelligence was assessed using a five-item scale crafted by Salovey and Mayer (1990), which includes phrases like “I easily recognize my emotions as I experience them.” Psychological safety was assessed using items designed to measure the degree to which respondents felt safe to express their thoughts and share knowledge without fear of negative consequences. Psychological safety was evaluated with four items from a scale by Edmondson (1999), including statements like “Members of this team are able to bring up problems and tough issues.” Lastly, knowledge-sharing behavior was measured using items that evaluated the frequency and quality of respondents’ knowledge-sharing interactions with colleagues. Additionally, knowledge sharing behavior was measured using Huang (2009) scale, which contains three items, one of which is “I often share the reports and official documents from my work with the members of my team.”

Each of these scales retained their original format and used a 5-point Likert scale for responses, where 1 signifies “strongly disagree” and 5 “strongly agree.” The survey was provided to participants in both English and Chinese to ensure linguistic accessibility. The translation process involved a professional translating the survey into Chinese and another professional conducting a back-translation into English to verify the survey’s linguistic accuracy.

### 3.4 Data analysis

In this study, the proposed model was evaluated using the AMOS 26.0 software, leveraging the structural equation modeling (SEM) technique. SEM is a widely used method that is effective for assessing latent variables in measurement models as well as for testing hypotheses concerning latent variables in structural models.

## 4 Results

### 4.1 Measurement

This study assessed the internal consistency of the scale using both Cronbach’s alpha coefficient and the composite reliability (CR) coefficient. The results, presented in Table 2, show that the Cronbach’s alpha coefficient for all variables ranges from 0.874 to 0.963, indicating robust internal consistency. To demonstrate convergent validity, factor loadings were analyzed and are detailed in Table 2, with values ranging from 0.754 to 0.946 across all variables, supporting their convergent validity. Additionally, the convergent validity of the structure is corroborated by average variance extracted (AVE) values, all exceeding 0.6, as displayed in Table 2.

**Table 2:** Reliability and validity.

Items	Loadings	Cα	AVE	CR
**Psychological capital**		0.923	0.757	0.925
PC1	0.887			
PC2	0.910			
PC3	0.769			
PC4	0.907			
**Emotional intelligence**		0.963	0.839	0.963
EI1	0.901			
EI2	0.939			
EI3	0.877			
EI4	0.946			
EI5	0.915			
**Psychological safety**		0.874	0.640	0.876
PS1	0.767			
PS2	0.890			
PS3	0.754			
PS4	0.781			
**Knowledge sharing**		0.958	0.850	0.958
KS1	0.886			
KS2	0.939			
KS3	0.939			
KS4	0.923			

This study assessed discriminant validity by comparing the square roots of the AVE for each variable with its correlation coefficients with other variables. The findings, detailed in Table 3, reveal that the square roots of the AVEs for each variable are consistently higher than their respective correlations with other variables. This pattern indicates a strong discriminant validity for the variables used in the study.

**Table 3:** Discriminant validity.

Construct	1	2	3	4
Psychological capital (1)	0.870			
Emotional intelligence (2)	0.471**	0.916		
Psychological safety (3)	0.490**	0.611**	0.800	
Knowledge sharing (4)	0.588**	0.628**	0.547**	0.922

The square root of the AVE is in diagonals; off diagonals are Pearson’s correlations of constructs. **p < 0.01.

### 4.2 Structural model

The results from the analysis revealed significant correlations between the variables, confirming the study’s six hypotheses as outlined in Table 3. We employed structural equation modeling (SEM) for data analysis, assessing model fit using several indices, including the chi-square to degrees of freedom ratio (χ2/df), Goodness of Fit Index (GFI), Normed Fit Index (NFI), Comparative Fit Index (CFI), Tucker-Lewis Index (TLI), and Root Mean Square Error of Approximation (RMSEA). Specifically, the model fit indices were favorable (χ^2^/df = 1.949, GFI = 0.946, NFI = 0.971, CFI = 0.986, TLI = 0.983, RMSEA = 0.047), all within acceptable thresholds, suggesting a good fit of the model and enhancing our confidence in the study’s results.

**Figure 2:**
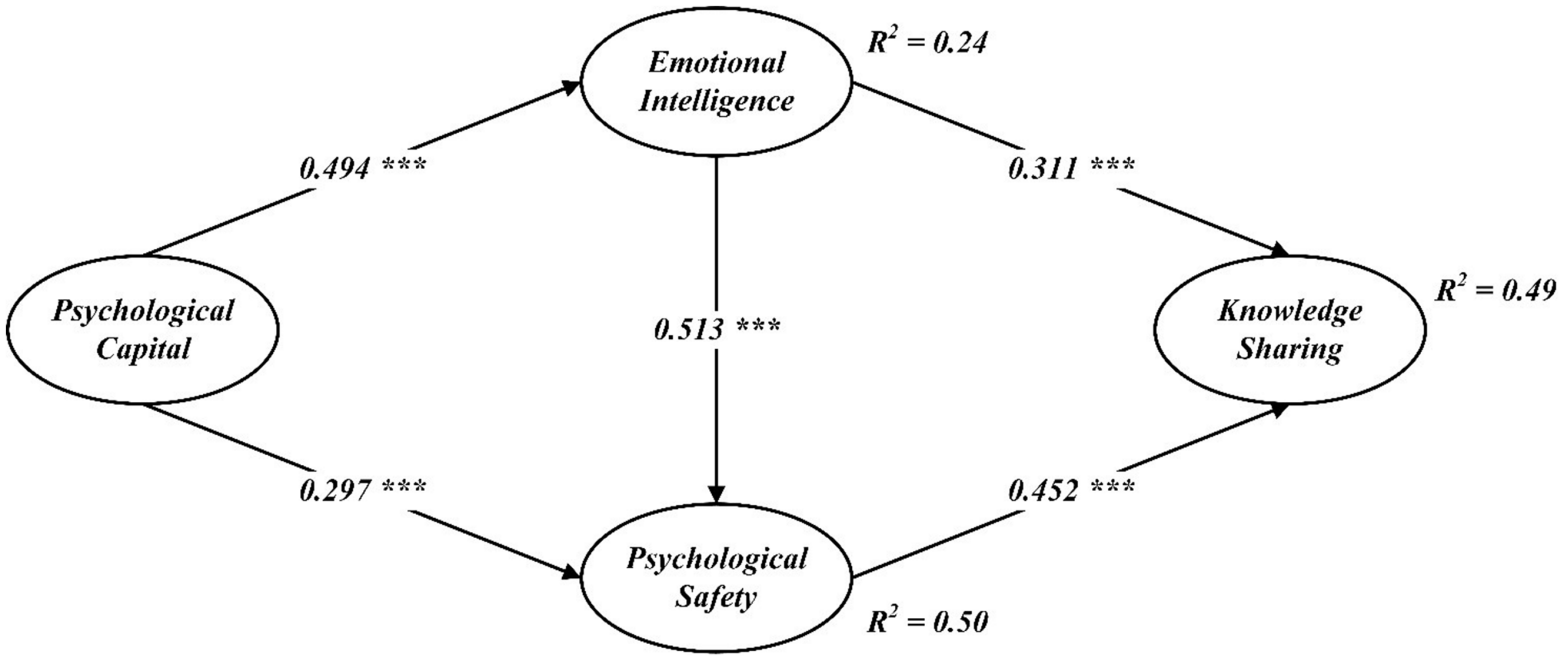
Structural model.

As depicted in Figure 2, the structural path model demonstrated significant positive correlations between psychological capital and both emotional intelligence (*β* = 0.494, *p* < 0.001) and psychological safety (*β* = 0.297, *p* < 0.001), thus confirming Hypotheses 1 and 2, respectively. Additionally, emotional intelligence showed a significant positive relationship with psychological safety (*β* = 0.513, *p* < 0.001) and knowledge sharing (*β* = 0.452, *p* < 0.001), supporting Hypotheses 3 and 4. Psychological safety was also found to be significantly positively correlated with knowledge sharing (*β* = 0.311, *p* < 0.001), affirming Hypothesis 5.

The mediating effects were examined using the bootstrap method, employing a 5,000-sample bootstrap with results presented within a 95% confidence interval in Table 4. The data illustrated that both emotional intelligence and psychological safety play significant roles in mediating the relationship between psychological capital and knowledge sharing, with a standardized indirect effect of 0.394 (*p* < 0.01), thus supporting Hypothesis 6.

**Table 4:** Indirect effects.

	Point estimate	Product of coefficients		Bootstrapping		
				Bias-corrected 95% CI		Two-tailed significance
		SE	*Z*	Lower	Upper	
PC → EI → PS → KS	0.394	0.043	9.163	0.311	0.477	*p* < 0.01

## 5 Discussion

### 5.1 Theoretical contributions

This study makes several significant theoretical contributions to the existing literature on organizational behavior, particularly within the context of e-commerce. This study makes several significant theoretical contributions to the existing literature on organizational behavior, particularly within the context of e-commerce. First, it extends the application of SET by integrating it with psychological constructs such as psychological capital, emotional intelligence, and psychological safety. While SET traditionally focuses on the exchange of tangible resources and the formation of trust and reciprocity (Schoenherr et al., 2015), this research highlights the importance of psychological resources in facilitating knowledge sharing. It demonstrates that employees’ psychological capital can significantly enhance their EI, which in turn fosters a culture of trust and collaboration, thereby promoting knowledge sharing.

Second, the findings extend the understanding of psychological capital’s impact by showing its positive influence on both emotional intelligence and psychological safety. Previous research has primarily focused on the direct effects of psychological capital on individual outcomes such as job satisfaction and performance (Peng et al., 2022). This study contributes to the literature by providing empirical evidence that psychological capital enhances not only emotional intelligence but also the sense of psychological safety among employee. This dual impact is particularly relevant for e-commerce practitioners who need to navigate complex emotional landscapes and feel secure in their work environment to share knowledge effectively.

Third, this study underscores the mediating roles of emotional intelligence and psychological safety in the relationship between psychological capital and knowledge sharing. While previous research has established the importance of emotional intelligence in facilitating interpersonal interactions and psychological safety in fostering open communication (He et al., 2019; Zulfadil et al., 2020), this research integrates these constructs to show their combined mediating effects. It reveals that psychological capital enhances emotional intelligence, which in turn improves psychological safety, ultimately leading to increased knowledge sharing. This mediation model offers a more nuanced understanding of how internal psychological resources translate into collaborative behaviors in the workplace.

Finally, the integration of emotional intelligence and psychological safety in this study provides a comprehensive framework for understanding the dynamics of knowledge sharing. While previous studies have examined these factors separately (Kim et al., 2015; Malik, 2022), this research shows that both emotional intelligence and psychological safety are essential for creating a supportive and collaborative organizational culture. High emotional intelligence facilitates better communication and trust, while psychological safety ensures that employees feel secure in sharing their knowledge. This dual focus offers a more holistic view of the factors that drive knowledge sharing in e-commerce settings.

In conclusion, this study integrates psychological capital, emotional intelligence, and psychological safety into the framework of SET, it provides a robust model for exploring the factors that drive effective knowledge sharing. These insights not only advance academic knowledge but also offer practical guidance for e-commerce organizations seeking to foster a culture of continuous learning and innovation.

### 5.2 Practical implications

The findings of this study provide actionable insights for e-commerce organizations aiming to enhance knowledge sharing among employees by leveraging psychological capital, emotional intelligence, and psychological safety. These implications can be divided into short-term, mid-term, and long-term strategies, all of which are grounded in the principles of SET, which emphasizes the importance of reciprocity and trust in fostering cooperative behaviors like knowledge sharing.

In the short term, e-commerce organizations should implement immediate actions based on the study’s findings that show a strong correlation between psychological capital and knowledge-sharing behaviors. Workshops and training sessions focusing on boosting employees’ psychological capital—particularly self-efficacy, hope, resilience, and optimism—should be rolled out. This will equip employees with the psychological resources necessary to cope with the fast-paced, high-pressure environment typical in e-commerce settings. The study suggests that these psychological traits are key drivers of emotional intelligence and psychological safety, both of which facilitate knowledge sharing. In addition to training, managers should be trained to identify and nurture these psychological attributes within their teams, creating a positive cycle that reinforces a collaborative and knowledge-sharing culture.

For the mid-term, the study underscores the importance of emotional intelligence as a mediator in knowledge sharing. Organizations should develop professional development programs aimed at enhancing employees’ emotional regulation, empathy, and communication skills. These attributes, as demonstrated by the research, are essential for fostering strong interpersonal relationships, which are critical for effective knowledge sharing. Practical steps include embedding emotional intelligence development into daily operations, such as performance evaluations and team-building activities, to ensure it becomes an integral part of the company culture. Furthermore, creating formal mentorship programs and peer support networks, where employees can exchange experiences and best practices, will reinforce the collaborative atmosphere needed for sustained knowledge sharing.

In the long term, the study highlights the necessity of establishing and maintaining a psychologically safe work environment. This involves fostering an organizational culture that promotes openness, inclusivity, and respect—elements identified in the study as essential for long-term knowledge sharing and innovation. Organizations should regularly assess their workplace climate through surveys and feedback mechanisms to ensure that psychological safety is sustained over time. Leadership development programs should prioritize cultivating leaders who demonstrate supportive behaviors, as this study shows that such leadership is crucial for creating environments where employees feel safe to share ideas and take risks without fear of negative repercussions. Over the long term, embedding these leadership behaviors into the organization’s ethos will help institutionalize knowledge sharing as a core value, driving continuous innovation and a competitive edge.

By implementing these short-term, mid-term, and long-term strategies based on the current study’s findings, e-commerce companies can create a sustainable environment where knowledge sharing flourishes, ultimately leading to enhanced organizational performance and innovation capacity.

### 5.3 Limitations

While this study provides meaningful insights, several limitations should be acknowledged. First, it relies on self-reported data, which can introduce common method bias and social desirability bias. Despite efforts to mitigate these issues through the use of anonymity and validated scales, the risk of biased responses remains. Future research could benefit from adopting a multi-method approach, integrating objective data or third-party evaluations to provide a more accurate and balanced perspective.

Moreover, the cross-sectional design of this study limits its ability to establish causal relationships between psychological capital, emotional intelligence, psychological safety, and knowledge sharing. As such, it cannot fully capture how these variables interact and influence one another over time. To address this, future studies should adopt longitudinal designs that track changes in these constructs, providing clearer evidence of causality and how these dynamics evolve in real-world settings.

Lastly, this study does not account for other potential moderating and mediating factors that could affect the relationship between the studied variables, such as organizational culture, leadership styles, or technological infrastructure. Future research should explore these additional factors to offer a more comprehensive understanding of knowledge sharing in e-commerce and other sectors, potentially uncovering new pathways to enhance collaboration and innovation within organizations.

## 6 Conclusion

This study has explored the impact of psychological capital, emotional intelligence, and psychological safety on knowledge sharing behaviors among e-commerce practitioners, framed within the context of SET. The findings confirm that psychological factors play a crucial role in fostering knowledge sharing, highlighting the importance of building psychological capital and emotional intelligence to enhance collaboration and innovation within organizations. Additionally, the study demonstrates that psychological safety significantly contributes to creating a supportive work environment where employees feel encouraged to share knowledge without fear of judgment or negative repercussions. Through the application of SET, this study provides valuable empirical evidence that supports the relationships between the psychological variables and knowledge sharing, addressing a gap in the existing literature. It shows that employees are more likely to engage in knowledge sharing when they perceive psychological safety and possess strong psychological resources, validating the role of psychological capital and emotional intelligence in this process. The research findings also offer practical implications for e-commerce organizations, suggesting that fostering these psychological attributes can enhance overall organizational performance and sustain competitive advantage. In conclusion, this research contributes both theoretically and practically to the fields of organizational behavior and knowledge management within e-commerce. Future studies should further investigate the long-term effects of these psychological factors and consider additional variables such as organizational culture and leadership style to deepen the understanding of knowledge-sharing mechanisms.

## Data Availability

The original contributions presented in the study are included in the article/supplementary material, further inquiries can be directed to the corresponding author.
